# Role of Obesity in Asthma Control, the Obesity-Asthma Phenotype

**DOI:** 10.1155/2013/538642

**Published:** 2013-04-24

**Authors:** Shannon Novosad, Supriya Khan, Bruce Wolfe, Akram Khan

**Affiliations:** ^1^Pulmonary and Critical Care Medicine, Oregon Health and Science University, 3181 S.W. Sam Jackson Park Road, UHN67, Portland, OR 97239-3098, USA; ^2^Oregon Health and Science University, Portland, OR, USA

## Abstract

Asthma is a disease with distinct phenotypes that have implications for both prognosis and therapy. Epidemiologic studies have demonstrated an association between asthma and obesity. Further studies have shown that obese asthmatics have poor asthma control and more severe asthma. This obese-asthma group may represent a unique phenotype. The mechanisms behind poor asthma control in obese subjects remain unclear, but recent research has focused on adipokines and their effects on the airways as well as the role of oxidative stress. Both surgical and nonsurgical weight loss therapy have shown promising results with improvements in asthma control and decreased asthma severity. Comorbid conditions such as gastroesophageal reflux disease and obstructive sleep apnea may also have a role in poor asthma control in obese asthmatics. Further research is needed to define the mechanisms behind this phenotype which will guide the development of targeted therapies.

## 1. Introduction to the Obese-Asthma Phenotype

Obesity and asthma are major public health problems affecting large numbers of individuals across the globe. Obesity is often classified using body mass index (BMI) (Table 1) [1]. Worldwide obesity has more than doubled since 1980. In 2008, more than 1.4 billion adults, ≥20 years, were overweight. Of these, over 200 million men and nearly 300 million women were obese. It is estimated that at least 2.8 million adults die each year as a result of being overweight or obese [2].

The World Health Organization estimates that 235 million people currently suffer from asthma and that asthma is under diagnosed and undertreated [3]. Asthma prevalence (the percentage of people who have ever been diagnosed with asthma and still have asthma) increased from 7.3% in 2001 to 8.4% in 2010 in the United States [4]. In 2010, an estimated 25.7 million people had asthma: 18.7 million adults aged 18 and over, and 7.0 million children aged 0–17 years [4].

Both cross-sectional epidemiologic investigations and prospective studies have shown an association between asthma and obesity with a relative risk (RR) of up to 3.0 [5–8]. A meta-analysis of seven prospective studies showed an increased odds ratio (OR) for incident asthma of 1.92 (1.43–2.59) in those with obesity versus normal weight and concluded that the odds of incident asthma increased by 50% in overweight/obese individuals [9]. There was a dose-response relationship between body weight and asthma with increasing odds of incident asthma as BMI increased (*P* < 0.0001 for trend) [9]. Recent prospective studies have confirmed these earlier findings [10]. Studies have also shown an association between increased BMI and asthma in women as opposed to men suggesting that there may be sex-specific differences in the association between asthma and obesity [11, 12]. This association has however not always been borne out as some studies did not find a significant effect by sex [9].

The American Thoracic Society workshop in 2010 concluded that “asthma in the obese may represent a unique phenotype of asthma, with more severe disease that does not respond as well to conventional therapy” [13]. There is ongoing research to discover the etiology of this relationship and further define a distinct obesity-asthma phenotype. This review will focus on the relationship between obesity and asthma and the most current evidence regarding an “obesity-asthma” phenotype which is thought to have worsened asthma control and severity as well as a differential response to medications. 

## 2. Obese-Asthma Phenotype: Increased Severity and Decreased Control

In the United States, asthma remains inadequately controlled in up to 41–55% of patients [14, 15]. Identifying risk factors for uncontrolled asthma and using these risk factors to develop interventions is an active area of research. These identified factors can help define a specific phenotype. Asthma phenotypes previously identified include allergic, occupational, exercise-induced, nocturnal, aspirin-sensitive, and severe asthma [13]. Increased asthma severity and poor asthma control are characteristics of the obese-asthma phenotype.

Asthma control is defined in terms of both impairment and risk [16]. Impairment is the frequency and intensity of symptoms as well as the functional limitations a person experiences. It is measured using various validated questionnaires such as the Asthma Control Test (ACT) or the Asthma Control Questionnaire (ACQ) [16, 17]. Risk is determined by the possibility of future adverse events such as exacerbations and hospitalizations [16]. Severity can refer to a spectrum of findings including loss of function of the organs from asthma or to the occurrence of severe acute exacerbations [18].

Epidemiologic studies evaluating a number of risk factors and their association with asthma control have shown a significant association between obesity and poor asthma control (Table 2). Schatz et al. used the ACT questionnaire to examine factors associated with asthma control in 570 patients aged 35 years and older enrolled in a large managed healthcare organization [19]. In a multiple linear regression analysis, a higher BMI was an independent predictor of poor asthma control (*P* = 0.01). Demoly et al. studied 2337 Europeans with self-reported physician diagnosis of asthma and used the ACT questionnaire to measure asthma control. 30% of those with poor asthma control versus 22.7% with well controlled asthma (*P* < 0.001) had BMI greater than or equal to 30 kg/m^2^ [20]. Laforest et al. examined 1282 French patients identified by pharmacists (having at least one antiasthma medication prescribed), and multivariate analysis showed that BMI greater than 30 kg/m^2^ was an independent predictor of poor asthma control (OR 1.72 [1.11–2.65]) [21]. Stanford et al. enrolled 2238 outpatient adults and 2429 children across the United States and found that BMI greater than 30 kg/m^2^ was a predictor of uncontrolled asthma in adults but not in children (OR 1.54) [18]. 

Other studies have looked more specifically at obesity itself as a risk factor or attempted to define the association between obesity and asthma control/severity in a specific population. The majority of the studies have confirmed an association between obesity and worsened asthma control [22–26], quality of life [23, 25, 27], and/or severity [25, 28–30] (Table 2). 

Mosen et al. looked at both asthma control and severity using the Asthma Therapy Assessment Questionnaire (ATAQ), self-report of the number of asthma-related hospitalizations in the previous year, and Juniper mini-Asthma Quality of Life Questionnaire (AQLQ) [25]. They found that after adjusting for demographics, smoking status, oral corticosteroid use, GERD, and inhaled corticosteroids, a BMI ≥30 kg/m^2^ was significantly associated with poor asthma control (OR 2.7), poor asthma-specific quality of life (OR 2.8), and history of asthma-related hospitalizations (OR 4.6). Similarly in a Canadian outpatient population, higher BMI was associated with worsened asthma control and quality of life but not asthma severity [23]. Taylor et al. found that obese subjects with asthma were more likely to report continuous symptoms, miss work days, and use short-acting beta agonists and inhaled corticosteroids and were more likely to have severe persistent asthma [29]. Weight gain can lead to worsening asthma control or inability to gain control of asthma. Haselkorn et al. showed that asthma patients who gained five or more pounds over a 12-month period were more likely to have worsened asthma control and quality of life as well as the need for more steroid bursts than those patients who maintained their weight [31].

Most studies have shown worsened asthma control, quality of life, and/or severity in obese asthmatics; however, some studies have not found a significant association (Table 2) [32, 33]. A group of 292 people with asthma were recruited from five community-based outpatient primary care centers in urban settings, and asthma control was evaluated using four different validated asthma control questionnaires. They did not find any significant association between obesity and asthma control using the four different questionnaires. This finding persisted after adjusting for FEV1 (forced expiratory volume in one second), smoking status, race, sex, selected comorbid illnesses, and long-term asthma controller use [33]. Sastre et al. used the ACQ, ACT, and physician perception of control to evaluate asthma control in 607 Spanish adults. They did not find a significant association between BMI and asthma control but did find that the percentage of patients with poor control was slightly greater in patients with low BMI (<18.5 kg/m^2^) and high BMI (≥30 kg/m^2^) [32]. The lack of association in these studies compared to the majority of other studies could be due to differences in baseline asthma control and the percent of obese patients in the study groups. It is not clear at this time if obesity is associated with increased mortality in asthmatics. 

## 3. Obese-Asthma Phenotype: Response to Medications

The response to asthma medications may be influenced by obesity. Studies have shown differential responses to therapy in overweight or obese asthmatics compared to those with normal BMI (Table 3). Peters-Golden et al. performed a post hoc analysis of more than 3000 subjects with moderate asthma who were enrolled in 4 different randomized controlled trials [34]. The trials randomized patients to montelukast, beclomethasone, or placebo. The primary end point was asthma control days (ACD), and other end points included FEV1, beta-agonist use, and night-time awakenings. The placebo response for all end points was generally lower with increasing BMI. For ACD, there was a decrease in the response to the inhaled corticosteroid (beclomethasone) with increasing BMI, whereas the response to the leukotriene antagonist (montelukast) remained stable [34]. Boulet and Franssen found that obese individuals do not respond as well to an inhaled corticosteroid or inhaled corticosteroid/long-acting bronchodilator combination [35]. Farrah et al. examined asthma control after treatment with inhaled corticosteroid and found similar improvements across BMI groups in spirometry, airway inflammation, and airway hyperresponsiveness. In this study there was still an association with ACQ scores and BMI after treatment suggesting an effect of obesity on asthma independent of airway inflammation [36]. Sutherland et al. found decreased in-vitro response to glucocorticoids in obese asthmatics [37]. In this study, immune cells derived from both the peripheral blood and lungs showed reduced induction of mitogen-activated protein kinase phosphatase-1 (MKP-1) expression in response to dexamethasone in overweight/obese asthmatics compared to normal weight asthmatics [37]. The trial “The Study of the Effectiveness of Low Dose Theophylline as Add-On therapy in Poorly Controlled Asthma” or “LODO” enrolled 488 subjects and compared theophylline, montelukast, and placebo in subjects with mild-moderate asthma who were not controlled on their current therapy. This group of patients was categorized by Dixon et al. as normal weight, overweight, and obese according to BMI. They found that obese patients who took theophylline had a trend towards an increased rate of exacerbations compared to placebo (8.1 versus 4.8 events per year, *P* = 0.06) and the relative risk for exacerbation associated with obesity among patients on theophylline was 3.7 (*P* < 0.001). There were no significant differences in response to treatment with montelukast [38]. 

## 4. Obese-Asthma Phenotype: Effect of Weight Loss

In general, studies have shown improvement in various parameters of asthma control and severity with weight-loss interventions ranging from diet modification to bariatric surgery. Juel et al. published a systematic review of weight-loss interventions in 2012 and concluded that all papers reviewed showed some positive effect of weight loss on asthma control [39]. 

## 5. Obese-Asthma Phenotype: Nonsurgical Weight Loss Interventions

In an open, randomized parallel group study, Stenius-Aarniala et al. investigated the effects of a supervised weight reduction program (eight-week, very low-energy diet) and found significant improvements in FEV1, forced vital capacity (FVC), dyspnea, the use of rescue medications, and the number of exacerbations [40]. An uncontrolled study which included 24 obese women with asthma enrolled in a program consisting of a 900 kcal per day diet and regular exercise found improvements in FEV1, FVC, and total lung capacity (TLC) but no significant improvements in airway responsiveness (defined as responsiveness to inhaled methacholine) [41]. 

In a recent Cochrane review, Adeniyi and Young assessed the effect of various weight-loss interventions on asthma control [42]. They included four studies with total of 197 adults. Interventions included supervised physical activity, low calorie diet, and antiobesity drugs. They decided that there were methodological flaws in the studies with an unclear risk of selection bias, high risk of detection bias, and small sample size. They concluded that the benefit of weight loss remains uncertain and there is a need for more randomized controlled trials.

## 6. Obese-Asthma Phenotype: Bariatric Surgery and Asthma Control

In 1999, Dixon et al. evaluated 32 patients with asthma twelve months after Lap-Band surgery [43]. Mean BMI was 46 kg/m^2^ prior to surgery and 33 kg/m^2^ at follow-up. Significant improvements were seen in severity, daily impact, medications needed, hospitalization, sleep, and exercise. Eleven patients reported no longer having asthma symptoms. Maniscalco et al. compared twelve obese asthmatic females who underwent laparoscopic adjustable gastric banding (LAGB) to ten nonoperated obese asthmatic females [44]. After surgery, those undergoing surgery had improved ACT scores compared to the group without surgery (18.7 to 22.2, *P* < 0.001). Shortness of breath and rescue medication use were significantly improved after surgery. In a prospective study, Dixon et. al. compared 23 asthmatic (mean BMI 51 kg/m^2^) and 21 nonasthmatic (mean BMI 38 kg/m^2^) patients undergoing bariatric surgery [45]. At baseline, asthmatic patients had a lower FEV1 and FVC. Twelve months after surgery the asthmatic patients experienced significant improvements in asthma control (asthma control score 1.55 to 0.74, *P* < 0.0001) and asthma quality of life (4.87 to 5.87, *P* < 0.0001). In addition, they had a significant improvement in airway responsiveness to methacholine but no significant changes in inflammatory markers [45]. 

## 7. Potential Mechanisms of Interaction between Asthma and Obesity

The association between asthma and obesity is well established, and obese asthmatics have been shown to have worsened asthma control and increased asthma severity. The mechanisms behind this are still under investigation and likely multifactorial. This has made it more difficult to measure the impact of obesity on objective asthma traits such as the measurement of specific inflammatory mediators or airway hyperreactivity.

## 8. Atopy and Eosinophilic Airway Inflammation

Eosinophilic airway inflammation is a characteristic of atopic asthma. Studies have found that the relationship between obesity and asthma is stronger in nonatopic individuals [47–50]. Appleton et al. looked at the association between asthma and obesity as measured by waist circumference (WC) and waist-to-hip ratio (WHR) and whether or not atopy modified this relationship. They used skin prick tests to a panel of allergens to define atopic status. When they stratified according to atopic status, they found that the relationship between obesity and asthma was significant only in nonatopic individuals when using WC and WHR as a marker of obesity [48]. Chen et al. examined the association between obesity and asthma in both atopic and nonatopic individuals. Allergy skin tests were used to measure atopic status. In nonatopic subjects, the odds ratio for asthma with a BMI of at least 30 kg/m^2^ was 2.01 (95% CI 1.13, 3.59). The association between asthma and obesity was not statistically significant in atopic subjects [49]. Fenger et al. explored the association between adiposity (using six different measurements) and asthma. They found that all adiposity measurements were associated with a higher prevalence of asthma but only among nonatopic individuals [50].

Fraction of exhaled nitric oxide (FeNO) is used as a marker of airway inflammation and as an indirect measurement of the presence of airway eosinophils [51, 52]. Studies have not shown a relationship between obesity and eosinophilic airway inflammation [53]. Others have shown an inverse relationship between sputum eosinophils and waist circumference in asthmatics [24]. Todd et al. found no significant differences in sputum eosinophil counts between obese and nonobese asthmatics [54]. Decreased levels of FeNO have been found in obese individuals who report wheezing [55]. Scott et al. found that obesity had no effect on sputum eosinophil counts in asthmatics, but obese asthmatics did have a higher sputum neutrophil percentage [56]. These findings suggest that obesity is not associated with increased airway eosinophilic inflammation and that allergic inflammation cannot explain the association between obesity and asthma.

## 9. Leptin and Adiponectin 

Obesity is characterized by chronic low-grade systemic inflammation. Obese adipose tissue is infiltrated by macrophages that are a source of inflammatory cytokines [57]. TNF-alpha, Il-6, and leptin have been shown to be higher in obese asthmatics patients than in nonobese asthmatics [58]. More than 50 different adipokines are secreted by adipocytes. Adipokines are proteins that help regulate various body functions [59].

Leptin and adiponectin are two adipokines that are being studied to determine their association with asthma. Serum leptin is proinflammatory, and serum levels are markedly increased in obesity. Serum adiponectin is lower in obesity and has important antiinflammatory effects in obesity [59]. 

Shore et al. showed that giving exogenous leptin to sensitized mice augmented airway hyperreactivity after allergen challenge [60]. Lugogo et al. examined 42 subjects with asthma and 46 healthy controls [61]. They found that leptin levels were increased in overweight/obese subjects regardless of asthma status (*P* = 0.013) but were significantly higher in those with asthma. Levels of TNF-alpha were also higher in overweight/obese subjects with asthma [61]. Alveolar macrophage response to bacterial lipopolysaccharide (LPS) was most robust in overweight/obese subjects with asthma. Preexposure to high-dose leptin enhanced proinflammatory response. Leptin alone induced production of proinflammatory cytokines from macrophages derived from overweight/obese subjects with asthma showing that macrophages from overweight/obese people with asthma are more sensitive to leptin. In another study, a cohort of obese women with and without asthma were followed for 12 months after gastric surgery [62]. Those with asthma had increased macrophage infiltration of visceral adipose tissue (*P* < 0.01), increased expression of leptin (*P* < 0.01), and decreased adiponectin (*P* < 0.001) when controlled for BMI [62]. Airway epithelial cells expressed receptors for leptin and adiponectin, and airway reactivity was significantly related to visceral fat leptin expression.

A bidirectional relationship between adiponectin and asthma has been noted in mice where allergen inhalation reduces serum adiponectin and exogenous adiponectin attenuates airway hyperreactivity [59]. Sood et al. showed a protective association between high serum adiponectin and odds for clinical diagnosis of asthma in premenopausal women independent of BMI and later showed that low serum adiponectin was predictive of future incident asthma [63, 64]. Sutherland et al. were not able to confirm these findings in 1000 32-year-olds in New Zealand. They found that higher serum adiponectin concentrations were associated with increased prevalence of reversible airflow obstruction and lower levels of exhaled nitric oxide among men suggesting a proinflammatory effect among men [65].

## 10. Lung Function

Obesity has an effect on lung function independent of its effect on asthma which may contribute to the poor control and increased severity of asthma in this phenotype. Functional residual capacity (FRC) and expiratory reserve volume (ERV) have been consistently shown to decrease with increasing BMI [66, 67]. At a BMI of 30 kg/m^2^, FRC and ERV are only 75% and 45% of the values seen in those with BMI of 20 kg/m^2^ [66]. The changes in TLC associated with obesity are smaller, and TLC is usually well preserved until a patient becomes markedly obese [66, 68]. Obesity is not typically associated with airflow obstruction, and the FEV1/FVC ratio is preserved [66, 69]. Obese patients have increased airway resistance. This could be at least partially explained by the fact that they breathe at a lower FRC leading to closure of smaller airways [68, 70]. The distribution of adipose tissue (upper versus lower body) may be the most important determinant of altered lung mechanics [71].

Obesity may have an effect on airway smooth muscle leading to increased airway hyperreactivity but the current evidence is conflicting. A study of non-obese, asthmatic subjects showed increased response when they had an acute reduction in lung volumes [72]. Salome et al. found no significant differences in the response to methacholine in obese and non-obese subjects [73]. They did find that with bronchoconstriction, obese subjects develop increased elastic loads which could lead to greater perception of dyspnea. A study evaluating the dynamic changes in lung volumes before and after methacholine challenge found that dynamic changes with bronchoconstriction were greater in obese than nonobese asthmatics [74]. 

## 11. Oxidative Stress

Increased oxidative stress either systemically or in the airways has been studied in association with both asthma and obesity. Higher serum F2-isoprostane levels were associated with asthma, but the association was not significant after adjusting for obesity or gender [75]. Another study showed an association between exhaled 8-isoprostane and BMI in asthmatics only [76]. Exhaled and serum 8-isoprostane were measured in a group of moderate-to-severe asthmatics and controls. Obesity was associated with increased exhaled 8-isoprostane in both asthmatics and controls, while plasma levels were higher in asthmatics than in controls. There was no correlation between the serum and exhaled levels [77]. At present, it is difficult to know what role oxidative stress plays in the obese-asthma phenotype. 

## 12. Obese-Asthma Phenotype: Role of Comorbid Conditions 

Obesity is associated with a number of comorbid conditions that may either contribute to the observed obese-asthma phenotype or may themselves be associated with asthma. These include gastro-esophageal reflux disease (GERD) and obstructive sleep apnea (OSA).

GERD is frequently cited as a reason for uncontrolled asthma in both the literature and in clinical practice. Obese patients are frequently bothered by GERD. A recent meta-analysis that included eleven trials covering 2524 patients found that patients had a higher mean morning peak expiratory flow (PEF) rate after treatment with proton pump inhibitory (PPI) but no significant difference in asthma symptoms score, asthma quality of life questionnaire, evening PEF rate, and FEV1. Overall they found insufficient evidence to recommend empirical use of PPIs for routine treatment of asthma [78]. These results conflict with current clinical practices and overall lead to the question of whether treating GERD would improve asthma control regardless of BMI.

Julien et al. performed an overnight home polysomnography on 26 patients with severe asthma, 26 patients with moderate asthma, and 26 controls without asthma of similar age and BMI. They measured flow rates as well as asthma control and quality of life. They found that OSA was present in 50% of the subjects with severe asthma, 23% with moderate asthma, and 12% of controls (*P* = 0.007) [79]. They also found that apnea-hypopnea severity measures were significantly worse for both asthmatic groups compared to controls. They did not find any significant correlation between severity of sleep-disordered breathing and asthma severity or control measures. Dixon et al. looked at both GERD and OSA in a group of asthmatic patients participating in a trial of reflux treatment [80]. They found that witnessed apnea had the strongest association with decreased asthma control and that there was a highly significant relationship between the number of symptoms of OSA reported by subject and asthma control [80]. They found no relationship between GERD and asthma control in obese patients.

Teodorescu et al. studied 472 patients with asthma who completed the Sleep Apnea Scale of the Sleep Disorders Questionnaire (SA-SDQ) and ACQ [81]. High OSA risk was associated with 2.87 times higher odds of poor asthma control (95% CI, 1.54–5.32; *P* = 0.0009) after adjustment for obesity and other factors [81]. 

OSA has been shown to worsen nocturnal asthma [82]. Teodorescu et al. looked at the association between OSA and daytime asthma in a group of asthmatic patients at tertiary-care centers. Subjects completed the SA-SDQ, and medical records were reviewed for established diagnosis of OSA and CPAP use. After using modeling to control for obesity and other risk factors, they found that high OSA risk was associated with persistent daytime (OR = 1.96 [1.31–2.94]) and nighttime symptoms (1.97 [1.32–2.94]). A diagnosis of OSA was associated with persistent daytime symptoms (2.08 [1.13–3.82]) but not nighttime symptoms (1.48 [0.82–2.69]). Using CPAP lowered the risk of persistent daytime symptoms (0.46 [0.23–0.94]). They concluded that both questionnaire-defined OSA risk and historical diagnosis were associated with persistent daytime asthma symptoms equal to or greater than the association with nighttime asthma symptoms [83].

## 13. Recommendations

Asthma and obesity exhibit a well-established relationship with evidence pointing towards a more severe and difficult-to-control obese-asthma phenotype that has altered responses to controller medications. This phenotype is more likely to have a worse quality of life, more daily symptoms, and more exacerbations as well as use more rescue medications. 

Research is underway to better define the mechanisms responsible for this distinct phenotype, which will allow for targeted therapies. The increased risk in this phenotype may be due to changes in inflammation from altered levels of leptin and adiponectin, increased oxidative stress, and/or obesity induced changes in lung volumes. The authors recommend that due to the varying response to controller medications, which makes it difficult to know which medication regimen is ideally suited to treat this phenotype, clinicians taking care of patients with asthma should follow the obese asthmatic closely to assess response to medications. It is possible that obese asthmatics may not respond as well to corticosteroids as compared to individuals of normal weight. Rather than increasing doses of medications when an obese patient does not respond as expected and increasing the risk of side effects, the clinician could focus on alternative methods of disease management. Besides medications to control asthma, weight management programs and early detection and management of OSA may offer targeted therapies that can improve asthma control. Weight loss interventions, both surgical and nonsurgical, can improve asthma control in the appropriate candidate. Efforts focused on prevention of obesity may be the most beneficial therapy for this phenotype.

## Figures and Tables

**Table 1:** WHO body mass index (BMI) Classification [1].

Classification	BMI (kg/m²)	Risk of comorbidities
Underweight	<18.5	Low (but risk of other clinical problems increased)
Normal range	18.5–24.9	Average
Overweight (preobese)	25.0–29.9	Mildly increased
Obese	≥30.0	
Class 1	30.0–34.9	Moderate
Class 11	35.0–39.9	Severe
Class 111	≥40.0	Very severe

**Table 2:** Studies of obesity as a risk factor for asthma control and severity.

Study	Study population	Number of subjects	Results
Barros et al., 2011 [22]	Patients with severe asthma in Brazil	508	Obese asthmatics had worse asthma control
Peters et al., 2011 [27]	Adult outpatients in South Texas	429	Obese asthmatics had worse quality of life and lower FVC but no difference in asthma severity and health care utilization
Youkou et al., 2011 [30]	Japanese outpatients	3146	Obese asthmatics had more severe disease and higher utilization of inhaled salmeterol and leukotriene receptor antagonists
Sastre et al., 2010 [32]	Spanish outpatients	607	No significant association between obesity and asthma control
Stanford et al., 2010 [18]	American outpatients	2238	Obesity was associated with worsened asthma control as measured by ACT.
Clerisme-Beaty et al., 2009 [33]	Urban outpatients in the United States	292	No association between asthma control and obesity
Demoly et al., 2009 [20]	European internet survey	2337	Obesity was associated with worsened asthma control as measured by ACT.
Haselkorn et al., 2009 [31]	Adult outpatients	2396	Worsened control of asthma with weight gain
Lessard et al., 2008 [24]	Adult outpatients in Canada	88	Obese asthmatics had worse asthma control
Mosen et al., 2008 [25]	Adult members of managed care organization	1113	Obese asthmatics had worse asthma-related quality of life, asthma control, and history of asthma related hospitalizations.
Taylor et al., 2008 [29]	Outpatient adults from 4 states	3095	Obese asthmatics had worse more severe asthma based on measurement of several different factors
Schatz et al., 2007 [19]	Adult members of managed care organization	570	Obesity was associated with worsened asthma control as measured by ACT.
Laforest et al., 2006 [21]	French outpatients	1282	Obesity was associated with worsened asthma control as measured by ACT.
Lavoie et al., 2006 [23]	Canadian outpatients	382	Obese asthmatics had worse asthma control and quality of life but not asthma severity
Saint-Pierre et al., 2006 [26]	French outpatients, from 4 university-based clinics	406	Overweight/obese asthmatics less likely to develop better control of asthma
Akerman et al., 2004 [28]	Adults outpatients in the United States	143	Linear relationship between asthma severity and BMI

**Table 3:** Studies of responses to therapy in overweight or obese asthmatics compared to normal BMI.

Study	Asthma therapy	Number of subjects	Results
Farah et al., 2011 [36]	Fluticasone/salmeterol	49	Similar improvements in asthma control, spirometry, airway inflammation, and airway hyperresponsiveness across BMI groups
Sutherland et al., 2010 [46]	Fluticasone, montelukast	1052	Fluticasone greater improvement across all BMI categories when compared to montelukast
Sutherland et al., 2008 [37]	Glucocorticoids	45	Decreased in vitro response to glucocorticoids in obese asthmatics
Boulet and Franssen 2007 [35]	Fluticasone with or without salmeterol	1242	Fluticasone with salmeterol resulted in improved asthma control in both groups compared to fluticasone alone but overall decreased effectiveness with both regimens in obese patients
Dixon et al., 2006 [38]	Theophylline, montelukast, placebo	488	Increased risk of exacerbations in obese subjects treated with theophylline but no difference in montelukast treatment groups
Peters-Golden et al., 2006 [34]	Beclomethasone, montelukast, placebo	3073	Less improvement in asthma control days with inhaled corticosteroid with increasing BMI, no difference with montelukast
